# Effectiveness of *Bacillus clausii* (O/C, N/R, SIN, T) in the Prevention of Antibiotic-Associated Diarrhea and Gastrointestinal Symptoms: A Systematic Review

**DOI:** 10.3390/antibiotics14050439

**Published:** 2025-04-27

**Authors:** Ana Teresa Abreu, Rodrigo Vázquez Frías, Christian Boggio Marzet, Juan Pablo Stefanolo, Alejandro Concha Mejía, Luis Bustos Fernández, Oscar Laudanno, Dimas Rosa, Maria Claudia Cruz Serrano, Karen Cárdenas, Julio Zuluaga

**Affiliations:** 1 Hospital Angeles del Pedregal, Mexico City 10700, Mexico; 2 Hospital Infantil de México Federico Gómez, Mexico City 06720, Mexico; rovaf@yahoo.com; 3 Grupo de Trabajo en Gastroenterología y Nutrición Pediátrica, Hospital Gral. de Agudos “Dr. I. Pirovano”, Buenos Aires C1428, Argentina; cboggio35@hotmail.com; 4 Neurogastroenterology Laboratory, Favaloro Foundation–University Hospital, Buenos Aires C1095, Argentina; 5 Clínica del Country, Clínica la Colina, Hospital Universitario Nacional de Colombia, Universidad Nacional de Colombia, Bogotá 111321, Colombia; 6 Centro Médico Dr. Bustos Fernández, Buenos Aires C1428, Argentina; 7 Instituto de Investigaciones Médicas Alfredo Lanari, Universidad de Buenos Aires, Buenos Aires C1427, Argentina; oscarlaudanno@gmail.com; 8 Grupo de Investigación del Caribe y Centroamérica para la Microbiota, Probióticos y Prebióticos GICCAMPP, Santa Marta 470001, Colombia; dimasrosa@gmail.com; 9 Opella, a Sanofi Company, Bogotá 110121, Colombia; 10 Odds Epidemiology, Bogotá 111321, Colombiagerenciamedica@oddsepidemiology.com (J.Z.)

**Keywords:** *Bacillus clausii*, antibiotic-associated diarrhea, probiotics, dysbiosis, systematic review

## Abstract

**Background**: Dysbiosis and antibiotic-associated diarrhea (AAD) are significant concerns in clinical settings. Probiotics, such as *Bacillus clausii* (O/C, N/R, SIN, T), a spore-forming bacterium resistant to gastrointestinal conditions and most commonly used antibiotics, emerge as a promising approach. We aim to assess the role of *B. clausii* in preventing AAD in children and adults during antibiotic therapy. **Materials and methods**: A systematic literature search was conducted across multiple databases, including MEDLINE, EMBASE, Cochrane Library, LILACS, and SciELO, up to May 2024. Studies were included if they involved *B. clausii* (O/C, N/R, SIN, T) administration during antibiotic treatment and reported AAD-related outcomes. **Results**: A total of four studies were included in the review. The studies comprised two randomized controlled trials (RCTs), one meta-analysis of RCTs, and one expert consensus. The primary outcome was the effectiveness of *B. clausii* (O/C, N/R, SIN, T) in reducing the incidence of diarrhea. Results showed a significant reduction in the risk of AAD and gastrointestinal symptoms in patients receiving it at a dosage of 4 × 10^9^ CFU/day for children and 6 × 10^9^ CFU/day for adolescents and adults for up to 14 days. **Conclusions**: *B. clausii* (O/C, N/R, SIN, T) appears to be an effective probiotic for preventing AAD in adults and children. It significantly improves gastrointestinal symptoms associated with antibiotic treatment, including diarrhea, nausea, and epigastric pain. Future studies are recommended to further elucidate its effectiveness in diverse populations, especially in low- and middle-income countries.

## 1. Introduction

The human gut microbiota plays a critical role in maintaining overall health by performing metabolic, nutritional, protective, and structural functions in the intestine, contributing to physical and mental well-being, among other functions [1]. Disruptions in the balance of this microbial community, known as dysbiosis, can result from and are associated with various factors/diseases, including dietary habits, inflammatory bowel disease, irritable bowel syndrome, celiac disease, allergies, asthma, metabolic syndrome, cardiovascular diseases, and obesity [2,3]. One of the most common factors associated with dysbiosis is the use of antibiotics and their potential impact on the gut microbiome, commonly referred to as antibiotic-associated dysbiosis (AAD). AAD can manifest from the first antibiotic dosage up until 2 months after the last dose and affects between 5% and 35% of individuals taking antibiotics; of these, *Clostridioides difficile* is responsible for 15% to 25% of cases [4,5]. Antibiotics, can disrupt the normal gut flora, weaken the intestinal barrier, and allow the colonization of pathogenic and opportunistic bacteria, leading to symptoms such as frequent and loose stools [6].

Despite the clinical significance of AAD, the research surrounding its prevention and management has largely centered on probiotics containing strains from the *Lactobacillus* and *Bifidobacterium* genera. These strains are well-documented for their ability to restore gut homeostasis by competing with pathogenic bacteria, enhancing intestinal barrier function, and modulating immune responses [7,8]. Numerous studies have demonstrated their efficacy in reducing the incidence and severity of AAD. However, this body of evidence has limitations, as it tends to focus on a narrow range of probiotic species, leaving gaps in our understanding of alternative probiotics that may offer similar or superior benefits, like *Saccharomyces Boulardii*, which has a widely acknowledged role in the prevention of AAD [9]. For instance, while *Lactobacillus* and *Bifidobacterium* are part of the natural gut microbiota, probiotics with these species are not always resilient in the face of environmental stressors such as bile salts and gastric acidity, which can reduce their effectiveness in vivo [10,11].

Furthermore, existing research on probiotics and AAD often overlook specific clinical scenarios, such as administering multiple antibiotics or treating complex infections like *Helicobacter pylori*, where patients may be at higher risk for gut dysbiosis [7,8]. The heterogeneity in study designs, patient populations, and probiotic strains makes it challenging to draw definitive conclusions about the best strategies for preventing AAD across different contexts. Most studies lack standardization in probiotic dosing, formulation, and duration of treatment, which contributes to inconsistencies in the reported outcomes. This variability underscores the need for more robust studies that explore a broader range of probiotics, particularly those with unique properties, such as *B. clausii*. *B. clausii* is a spore-forming probiotic with the ability to survive gastric acid and bile salts, which means that up to 100% of its colony-forming units (CFU) reach the gut alive. Among other properties, it has demonstrated production of riboflavin (vitamin B2) and adherence to the intestinal wall with increase in mucin secretion and reduction in gut permeability, production of lantibiotics like clausin and other antimicrobial peptides, and immunomodulatory properties [12,13], which may confer additional protection against the disruptions caused by antibiotic use, both during and after antibiotic therapy.

Given the increasing use of antibiotics and the potential gastrointestinal side effects associated with their consumption, there is a pressing need to explore alternative strategies to prevent AAD and improve patient outcomes. This study aims to bridge the gap in current knowledge by systematically reviewing the available evidence on the role of *B. clausii* (O/C, N/R, SIN, T) in the prevention and treatment of AAD. By assessing its probiotic effects and ability to alleviate antibiotic-related adverse events, we hope to provide further insights into the potential of *B. clausii* as an effective strategy for enhancing gut health and supporting the continued use of necessary antibiotic treatments.

## 2. Materials and Methods

### 2.1. Reporting Guidelines and Research Question

This systematic review adhered to the updated PRISMA 2020 guidelines, ensuring transparency and rigor throughout the review process [14].

The research question guiding this review was developed using the PICO framework. The population targeted included individuals from all age groups at risk of suffering from AAD, defined as diarrhea that occurs either during or after antibiotic use without any plausible explanation other than the administration of antibiotics. The intervention under consideration was the probiotic *B. clausii*, particularly *B. clausii* (O/C, N/R, SIN, T) with a dosage of 4 × 10^9^ for children and 6 × 10^9^ for adults. Comparisons included placebo treatments, other probiotic strains, and the absence of probiotics. The primary outcomes of interest were reduced adverse effects commonly linked to antibiotic use, such as diarrhea, stomach discomfort or abdominal pain, nausea, and vomiting.

### 2.2. Eligibility Criteria

Strict eligibility criteria were applied to ensure that the review focused on the most relevant evidence. Randomized controlled trials (RCTs) were prioritized due to their high methodological rigor and ability to minimize bias. However, in cases where RCT data were insufficient to fully address the research question, other study designs, including observational studies, case reports, or case series, were also considered. This flexibility allowed for the inclusion of diverse evidence, which could offer additional insights into the effects of *B. clausii* (O/C, N/R, SIN, T) on AAD. Full-text publications were preferred, although abstracts and pre-prints were considered to capture the most up-to-date research. The search included studies published in indexed journals and gray literature, with no restrictions on publication date. Only studies published in English or Spanish were included. Narrative reviews, study protocols, commentaries, and letters to the editor were excluded.

### 2.3. Information Sources

A comprehensive literature search was conducted across multiple electronic databases chosen for their broad coverage of biomedical research. These included MEDLINE (via PubMed), Embase (via Elsevier), the Cochrane Central Register of Controlled Trials, LILACS (via *Biblioteca Virtual de Salud*), SciELO, and Google Scholar. In addition to the database search, a manual search was conducted using snowballing. This method involved reviewing the reference lists of studies identified in the initial search to capture any additional relevant studies that may not have been found through electronic databases alone. The search terms were derived from the PICO framework, utilizing a combination of controlled vocabulary (such as MeSH and DeCS terms) and free-text keywords suggested by experts in the field. The search strategy was based on the keywords “*Bacillus clausii*” or “Enterogermina” to ensure the identification of publications related to *Bacillus clausii* (O/C, N/R, SIN, T) and then customized for each database, incorporating field-specific identifiers, truncation, and Boolean operators to maximize the sensitivity and specificity of the search (Appendix A).

### 2.4. Data Collection Process

All search results were exported in RIS format and managed using Rayyan, a web-based application designed for systematic review workflows [15]. Rayyan, a web-based and mobile application designed to streamline the process of conducting systematic reviews, particularly during the screening phase, also facilitated the removal of duplicate references, ensuring a reliable study selection process.

### 2.5. Study Selection

The process of selecting studies for inclusion was conducted in two phases. First, titles and abstracts were screened by two independent reviewers using Rayyan based on predefined eligibility criteria. This dual-review approach minimized bias and ensured that relevant studies were not excluded prematurely. Disagreements between reviewers were resolved through discussion, and if consensus could not be reached, a third reviewer was consulted to make the final decision. Following this initial screening, full-text articles from the remaining studies were retrieved for a more thorough evaluation. Again, two independent reviewers assessed the full texts, applying the same eligibility criteria to determine whether the studies should be included in the systematic review. This iterative, blinded, and paired approach ensured the robustness of the study selection process, as it reduced the potential for subjective bias in deciding which studies met the criteria for inclusion.

### 2.6. Risk of Bias Assessment

The Cochrane Risk of Bias (RoB 2.0) tool was used for RCTs to assess the methodological quality of the included studies [16]. The Risk of Bias in Non-randomized Studies of Interventions (ROBINS-I) tool was applied to observational studies [17]. Both tools are widely recognized and validated for assessing different research designs, providing a structured approach to evaluating potential sources of bias. Two independent reviewers conducted the assessments to ensure objectivity and consistency. Any disagreements in the risk of bias assessment were resolved through discussion between the reviewers. A third reviewer was involved in the decision-making process when consensus could not be reached. This rigorous assessment of bias was crucial for determining the reliability and validity of the included studies, ensuring that the review’s findings were based on high-quality evidence.

### 2.7. Data Extraction

To minimize errors, two researchers conducted the data extraction process independently, using a standardized template in Microsoft Excel. Extracted data included key study characteristics, such as title, authors, year of publication, journal, country, study design, and study period. Detailed information on the population was recorded, including age group, diagnosis, and the number of participants in both the intervention and control groups. Data on *B. clausii* dosage and frequency were extracted for the intervention, along with corresponding details for the comparator groups. Outcome measures were meticulously documented, including the measurement methods, follow-up duration, and results for each predefined outcome. Where available, effect estimates (e.g., odds ratios or relative risks) were extracted, along with confidence intervals or *p*-values. The risk of bias was also noted for each study. A second reviewer cross-verified all extracted data to ensure accuracy.

### 2.8. Data Synthesis

Due to the heterogeneity of the included studies, particularly in terms of population characteristics, interventions, and outcome measures, a meta-analysis was deemed inappropriate. Instead, a descriptive and qualitative synthesis of the findings was performed. This involved summarizing the results of individual studies and presenting them in a structured manner, focusing on key outcomes and study populations. The qualitative synthesis highlighted patterns and differences across the studies, providing a comprehensive overview of the evidence on the effects of *B. clausii* in preventing or reducing AAD. This approach allowed for a thorough examination of the data, even without statistical pooling, and offered valuable insights into the potential role of probiotics in managing antibiotic-related side effects.

## 3. Results

### 3.1. Search Results

**Figure 1:**
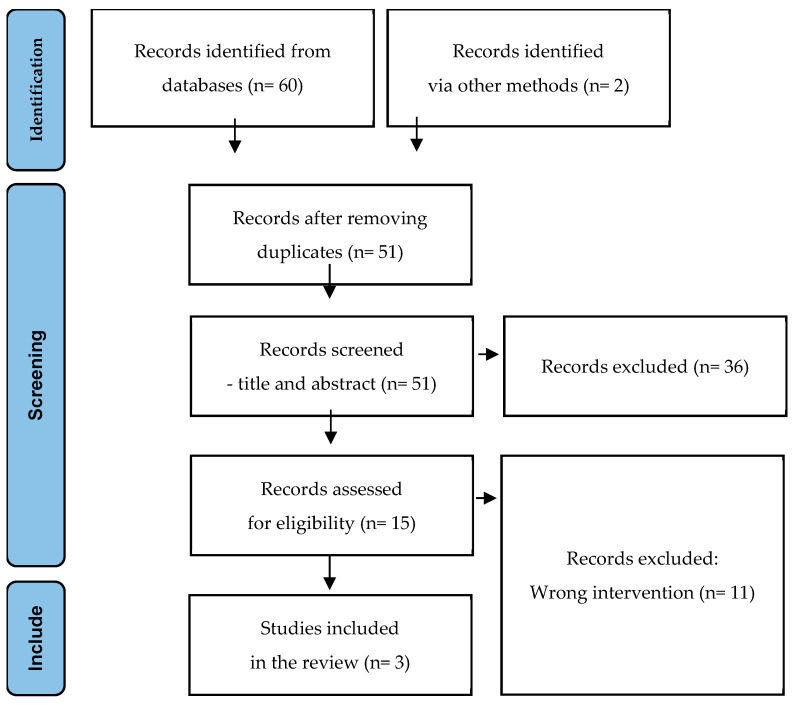
PRISMA 2020 flow diagram.

A total of 62 references were identified from the search across six electronic databases. After removing duplicates, 51 unique studies remained. These were screened by title and abstract, excluding 36 studies that did not meet the eligibility criteria. Subsequently, 15 full-text studies were assessed for inclusion. Of these, ten studies were excluded due to inappropriate intervention types. Four studies were included in this review: three randomized controlled trials (RCTs), one pooled analysis of RCTs, and one expert consensus. The expert consensus was then excluded, as it did not provide any original evidence regarding the research question and included recommendations of one of the included clinical trials. These studies formed the basis for the qualitative synthesis of the results. The PRISMA flow diagram (Figure 1) summarizes the selection process, indicating the studies included and excluded at each step.

### 3.2. Study Characteristics

The three included studies were published between 2004 and 2019 and involved populations ranging from 0.25 to 65 years old. Two studies focused on using *B. clausii* in antibiotic therapy for *Helicobacter pylori* eradication, while one examined various antibiotic regimens. In two studies, a placebo served as the comparator, and in one study, no probiotics were administered in the control group. All studies reported outcomes relevant to preventing or mitigating antibiotic-associated adverse effects. Detailed information on study characteristics is provided in Table 1.

### 3.3. Quality and Risk of Bias Assessment

The two RCTs were classified as having some concerns regarding the risk of bias. The risk of bias for each study is summarized in Table 2. The observational studies and consensus documents were evaluated for quality using the corresponding tools but were not the primary focus for bias evaluation.

### 3.4. Bacillus Clausii (O/C, N/R, SIN, T) Effects

#### 3.4.1. Adult Population

One of the included RCTs, conducted by Plomer et al. (2020) [18], evaluated the effects of *B. clausii* (O/C, N/R, SIN and T, 6 × 10^9^ CFU/day) in 130 adults receiving *H. pylori* eradication therapy in Italy. The study compared the probiotic to a placebo over two weeks. The eradication therapy consisted of clarithromycin 500 mg, amoxicillin 1 g, and rabeprazole 20 mg twice a day for one week. The mean age of the patients who received the intervention was 44.2 years (SD ± 13.5), and of the placebo group, it was 42.4 years (SD ± 13.0). After one week, the incidence of diarrhea in the intervention group was significantly lower than in the placebo group (29.2% vs. 47.7%; *p* = 0.030), with a 39% reduction in risk (RR 0.61; 95% CI 0.39–0.97). By the second week, the effect persisted in the per-protocol analysis (RR 0.25; 95% CI 0.07–0.84), though not in the intention-to-treat analysis (RR 0.38; 95% CI 0.14–1.02). The intervention group also reported a significant increase (*p* < 0.0304) in the number of days without diarrhea in week 1. Other side effects such as vomiting, nausea, and loss of appetite showed no significant differences between groups, except for epigastric pain, which was lower in the intervention group (RR 0.50; 95% CI 0.25–0.98). Adverse events were mild and unrelated to the probiotic.

A second RCT published in 2004 [19], also conducted in Italy, evaluated *B. clausii* (O/C, N/R, SIN and T, 6 × 10^9^ CFU/day) effects in 120 patients treated with the same *H. pylori* eradication therapy. The mean ages of the intervention and placebo group were 46.2 (SD ± 12.8) and 43.1 years (SD ± 13.4), respectively. This study found a reduction in nausea (RR 0.50; 95% CI 0.28–0.89) and diarrhea (RR 0.33; 95% CI 0.13–0.85) in the probiotic group during the first week, with effects on nausea persisting into the second week. The probiotic was well tolerated, with no significant adverse events reported.

#### 3.4.2. Pediatric Population

In a pooled analysis of three previously published RCTs, *B. clausii* (O/C, N/R, SIN, T) was evaluated in 435 children receiving antibiotic treatment (ampicillin, thiamphenicol, erythromycin, tetracycline, penicillin, or cephalosporin) for respiratory and urinary tract, tonsil, ear, and skin infections: 218 received antibiotic treatment with the probiotic, and 217 received antibiotic treatment alone (control group) [20]. It included studies published by Benoni et al. in 1984 [21] (n = 19; ages between 0.25 and 2 years), Puddu et al. in 1980 [22] (n = 93; ages 3–14 years), and Destura et al. in 2008 (unpublished data; n = 323; ages 0.5–12 years). The combined analysis demonstrated that the incidence of AAD was significantly lower in the probiotic group (1.8%) compared to the control group (6.5%) (*p* = 0.017). While individual studies did not show significant differences between groups, the pooled results provided more robust evidence in favor of *B. clausii* for preventing AAD in children.

#### 3.4.3. General Population

Considering the general population, an expert consensus from Asia in 2020 [23] recommended using *B. clausii* (O/C, N/R, SIN, T) as an adjunctive treatment for AAD prevention and as part of the *H. pylori* eradication therapy. The consensus highlighted that factors such as antibiotic type, treatment duration, hospitalization needs, age, comorbidities, and prior episodes of AAD should be considered when prescribing probiotics in pediatric patients. However, the consensus noted insufficient evidence to recommend *B. clausii* for *H. pylori* eradication in children.

## 4. Discussion

The prevention of AAD through administering *B. clausii* (O/C, N/R, SIN, T) has emerged as a promising intervention, evidenced by the findings from the studies included in this systematic review. The data consistently demonstrate that *B. clausii* significantly reduces the risk of developing AAD and alleviates other gastrointestinal symptoms, such as nausea and epigastric pain, particularly during the initial week of antibiotic therapy. Two RCTs indicate a considerable decrease in AAD incidence when *B. clausii* (O/C, N/R, SIN, T) is administered alongside antibiotics in adults receiving *H. pylori* eradication treatment. Similarly, pediatric populations, including infants and young children treated for various infections, also showed significantly reduced AAD incidence when supplemented with this probiotic. These findings underscore the clinical relevance of *B. clausii* (O/C, N/R, SIN, T) as a protective agent against AAD across a range of patient demographics.

The beneficial effects attributed to *B. clausii* (O/C, N/R, SIN, T) can be linked to its multifaceted mechanisms of action [13]. This probiotic strain induces immunomodulation, cytokine, immunoglobulin secretion, and water absorption [24,25,26], thus enhancing the integrity of the gut barrier. Further, natural resistance to several antibiotics is of great interest [27], particularly for O/C, N/R, SIN, and T strains that can tolerate different pH and oxygen conditions. However, considering that the concern about the development of antibiotic resistance usually arises when bacteria acquire resistance genes, often through plasmids—small pieces of DNA that can transfer genes between bacteria—since *B. clausii* (O/C, N/R, SIN, T) does not interact with or affect plasmids, it is unlikely to contribute to the generation or transfer of antibiotic resistance genes [28]. Probiotics are considered a valuable tool in the prevention of AAD. Meta-analyses of RCTs have concluded that probiotic strains have a moderate effect in their preventive role, both in children and adults [29], as well as in adults and the elderly [30]. The studies in this review, complemented by existing literature, support the notion that *B. clausii* (O/C, N/R, SIN, T) plays a critical role in maintaining gut health during antibiotic treatment by mitigating the disruption of the intestinal microbiota in adults [31] and children [32,33]. Moreover, the strain’s natural resistance to various antibiotics and adaptability to different pH and oxygen conditions further solidify its position as an effective therapeutic agent.

*B. clausii* (O/C, N/R, SIN, T) can help manage diarrhea and associated symptoms like nausea and abdominal pain, improving patient comfort during antibiotic therapy by fostering a favorable gut environment. Reducing these symptoms can also enhance treatment adherence, which potentially positively affects eradication rates and the reduction of antibiotic resistance. Although studies showing that probiotics improve *H. pylori* eradication rates are scarce, evidence indicates that better treatment adherence impacts eradication rates. There is also the rationale for using *B. clausii* (O/C, N/R, SIN, T) in combination with other probiotic strains like *Lactobacillus reuteri* DSM 17938 and ATCC PTA 6475 for eradication therapy, supported by the 2022 Maastricht VI/Florence guidelines.

All the studies evaluated the use of *B. clausii* (O/C, N/R, SIN, T) at a dosage of 4 × 10^9^ CFU/day for children and 6 × 10^9^ CFU/day for adolescents and adults for up to 14 days. These dosages align with recommendations from the World Gastroenterology Organization (WGO), which give evidence for use based on Oxford criteria and offer evidence based on publications with strain identity, colony-forming unit (CFU) quantity, and dose used in each study; they advocate for administering probiotics during antibiotic treatment and for seven days following its completion [9]. In studies related to diarrhea other than AAD, different dosages have been reported, such as 2 × 10^9^ CFU/day for children for five to seven days and 4 × 10^9^ CFU/day for adults over ten days. These findings suggest a probable need for higher doses in cases of AAD, where the gastrointestinal environment is significantly disrupted. However, it is noteworthy that there are currently no specific guidelines or recommendations for a standard dosage tailored for AAD prevention [23]. This lack of consensus emphasizes the need for further research to determine the optimal dosing strategies for *B. clausii* (O/C, N/R, SIN, T) in different clinical contexts, particularly in preventing AAD.

While the results are promising, several limitations must be considered when interpreting the findings of this review. The studies primarily originated from European countries, which may affect the generalizability of the results to populations in low- and middle-income countries. Additionally, although the included studies displayed some concerns regarding risk of bias, none were classified as high-risk. However, the relatively small number of RCTs in this review highlights the need for larger-scale studies to enhance the conclusions’ robustness. Variations in dosing across the studies raise additional questions about the optimal dosage of *B. clausii* required for effective prevention of AAD, especially when considering the different physiological needs of children and adults.

To address these limitations, future research should focus on conducting well-designed, large-scale RCTs in diverse populations, particularly in low- and middle-income countries where the burden of AAD is significant. Such studies should aim to clarify the efficacy of *B. clausii* (O/C, N/R, SIN, T) in various clinical settings and determine the optimal dosage for different age groups and underlying health conditions. Furthermore, exploring the long-term effects of *B. clausii* administration on gut health and overall well-being would be of great value. Addressing these questions will help elucidate the potential benefits of incorporating *B. clausii* into clinical practice as a preventive measure against AAD, ultimately contributing to enhanced patient care.

Evidence presented in this review supports the utilization of *B. clausii* (O/C, N/R, SIN, T) as an effective probiotic for preventing antibiotic-associated diarrhea in both adults and children. Its ability to mitigate gastrointestinal symptoms such as diarrhea, nausea, and abdominal pain highlights its clinical significance as an adjunct therapy during antibiotic treatment. Given the compelling data and all aforementioned properties, including mechanism of action, physiological properties, and antimicrobial and immunomodulatory activity, *B. clausii* (O/C, N/R, SIN, T) represents a promising approach to improving patient outcomes and minimizing the adverse effects of antibiotics on gut health. However, further research is necessary to confirm its efficacy across diverse populations and to refine dosage recommendations, ensuring optimal use of this probiotic in various healthcare settings.

## Figures and Tables

**Table 1:** Study characteristics, showing the detailed characteristics of the included studies.

Author, Year	Study Type	Population	Antibiotic Treatment	Intervention	Comparator	Outcomes	Main Results	Risk of Bias
Plomer, 2020 [18]	RCT	Adults (18–65 years)	H. pylori eradication: clarithromycin, amoxicillin, and rabeprazole for one week	*B. clausii* (O/C, N/R, SIN, T) (6 × 10^9^ CFU per day for two weeks) n = 65	Placebo (for two weeks) n = 65	Diarrhea	Week 1: RR 0.61; 95% CI 0.39–0.97 Week 2: RR 0.38; 95% CI 0.14–1.02	Some concerns
						Vomiting	Week 1: RR 2.00; 95% CI 0.38–10.54 Week 2: RR 1.00; 95% CI 0.14–6.89	
						Nausea	Week 1: RR 1.28; 95% CI 0.77–2.13 Week 2: RR 0.91; 95% CI 0.41–1.99	
						Epigastric Pain	Week 1: RR 1.03; 95% CI 0.71–1.51 Week 2: RR 0.50; 95% CI 0.25–0.98	
Nista, 2004 [19]	RCT	Adults (18–65 years)	H. pylori eradication: clarithromycin, amoxicillin, and rabeprazole for one week	*B. clausii* (O/C, N/R, SIN, T) (6 × 10^9^ CFU per day for two weeks) n = 60	Placebo (for two weeks) n = 60	Diarrhea	Week 1: RR 0.301; 95% CI 0.12–0.76 Week 2: RR 0.38; 95% CI 0.08–1.90	Some concerns
						Vomiting	Week 1: RR 0.78; 95% CI 0.22–2.71 Week 2: RR 0.48; 95% CI 0.09–2.52	
						Nausea	Week 1: RR 0.52; 95% CI 0.31–0.88 Week 2: RR 0.45; 95% CI 0.21–0.96	
						Epigastric Pain	Week 1: RR 0.68; 95% CI 0.48–0.97 Week 2: RR 0.86; 95% CI 0.51–1.47	
Plomer, 2019 [20]	Pooled analysis of three RCTs	Children (0.25–14 years)	Various antibiotics: ampicillin, tiamfenicol, erythromycin, tetracycline, penicillin, or cephalosporin	*B. clausii* (O/C, N/R, SIN, T) n = 218	No probiotics n = 217	Incidence of AAD	I = 1.8%, C = 6.5%; *p* = 0.017	Not applicable

**Table 2:** Risk of bias for the two RCTs included in this review.

Study (Author/Year)	Domain 1	Domain 2	Domain 3	Domain 4	Domain 5	Overall Risk of Bias
Plomer, 2020 [18]	Low	Low	Low	Some concerns	Some concerns	Some concerns
Nista, 2004 [19]	Low	Low	Low	Some concerns	Some concerns	Some concerns

## Data Availability

No new data was created or analyzed in this study.

## Appendix A

**Table A1:** Search strategies.

Search Type	Systematic
Database	**MEDLINE**
Platform	PubMed
Search Date	2 May 2024
Date Range	No restrictions
Language Restrictions	No restrictions
Other Limits	None
Search Strategy	#1 (Dysbiosis[Title/Abstract]) OR (Antibiotic-associated diarrhea[Title/Abstract]) 21131 #2 (bacillus clausii[Title/Abstract]) OR (Enterogermina[Title/Abstract]) 215 #3 #1 AND #2 16
References Identified	16
**Search Type**	**Systematic**
Database	**EMBASE**
Platform	ElSevier
Search Date	2 May 2024
Date Range	No restrictions
Language Restrictions	No restrictions
Other Limits	None
Search Strategy	(dysbiosis,ti OR “antibiotic associated diarrhea”,ti) AND “bacillus clausii”,ti OR enterogermina,ti 40
References Identified	40
**Search Type**	**Systematic**
Database	**Cochrane**
Platform	Cochrane Library
Search Date	2 May 2024
Date Range	No restrictions
Language Restrictions	No restrictions
Other Limits	None
Search Strategy	#1 MeSH descriptor: [Dysbiosis] explode all trees 221 #2 (“antibiotic associated diarrhea”),ab,kw 348 #3 #1 OR #2 569 #4 (“Bacillus clausii”),ab,kw 79 #5 (enterogermina),ab,kw 15 #6 #4 OR #5 81 #7 #3 AND #6 3
References Identified	3
**Search Type**	**Systematic**
Database	**LILACS**
Platform	Virtual Health Library
Search Date	2 May 2024
Date Range	No restrictions
Language Restrictions	No restrictions
Other Limits	None
Search Strategy	(dysbiosis) OR (antibiotic associated diarrhea) AND (Bacillus clausii) OR (enterogermina) 0
References Identified	0
**Search Type**	**Systematic**
Database	**SciELO**
Platform	Scientific Electronic Library Online
Search Date	2 May 2024
Date Range	No restrictions
Language Restrictions	No restrictions
Other Limits	None
Search Strategy	((((((ti:((ab:(((dysbiosis) OR (antibiotic associated diarrhea))))))))))) AND ((((((ti:((ab:(((Bacillus clausii) OR (enterogermina))))))))))) 0
References Identified	0
**Search Type**	**Systematic**
Database	**Google Scholar**
Platform	Google
Search Date	2 May 2024
Date Range	No restrictions
Language Restrictions	No restrictions
Other Limits	None
Search Strategy	allintitle: “dysbiosis” OR “antibiotic associated diarrhea” + “Bacillus clausii” OR “enterogermina” 1
References Identified	1
